# Vibration-Sensing Electronic Yarns for the Monitoring of Hand Transmitted Vibrations †

**DOI:** 10.3390/s21082780

**Published:** 2021-04-15

**Authors:** Zahra Rahemtulla, Theodore Hughes-Riley, Tilak Dias

**Affiliations:** Advanced Textiles Research Group, School of Art & Design, Nottingham Trent University, Bonington Building, Dryden Street, Nottingham NG1 4GG, UK; zahra.rahemtulla2018@my.ntu.ac.uk (Z.R.); tilak.dias@ntu.ac.uk (T.D.)

**Keywords:** electronic textiles, E-textiles, smart textiles, vibration, hand-transmitted vibration, health monitoring, occupational health

## Abstract

Overexposure to hand transmitted vibrations (HTVs) from prolonged use of vibrating power tools can result in severe injuries. By monitoring the exposure of a worker to HTVs, overexposure, and injury, can be mitigated. An ideal HTV-monitoring system would measure vibration were it enters the body, which for many power tools will be the palm and fingers, however this is difficult to achieve using conventional transducers as they will affect the comfort of the user and subsequently alter the way that the tool is held. By embedding a transducer within the core of a textile yarn, that can be used to produce a glove, vibration can be monitored close to where it enters the body without compromising the comfort of the user. This work presents a vibration-sensing electronic yarn that was created by embedding a commercially available accelerometer within the structure of a yarn. These yarns were subsequently used to produce a vibration-sensing glove. The purpose of this study is to characterize the response of the embedded accelerometer over a range of relevant frequencies and vibration amplitudes at each stage of the electronic yarn’s manufacture to understand how the yarn structure influences the sensors response. The vibration-sensing electronic yarn was subsequently incorporated into a fabric sample and characterized. Finally, four vibration-sensing electronic yarns were used to produce a vibration-sensing glove that is capable of monitoring vibration at the palm and index finger.

## 1. Introduction

This work demonstrates the creation and characterization of novel, textile, vibration-sensing electronic yarns (E-yarns), which have been incorporated into a vibration-sensing glove to create a proof-of-concept device for the monitoring of hand transmitted vibrations (HTVs). HTV exposure is frequently encountered in a number of professions, such as in the construction industry, where workers can experience prolonged exposure to significant levels of oscillatory motion (vibration) while working with hand-operated power tools. Overexposure can result in a variety of musculoskeletal, neurological, and vascular disorders [1,2] meaning that monitoring HTV exposure is important: Disorders include Hand Arm Vibration Syndrome (HAVS) and Carpal Tunnel Syndrome (CTS) [3].

The relationship between the use of vibrating tools and the manifestation of HAVS has been well documented in the literature [4,5,6]. Studies have shown that a decrease in the vibrotactile perception thresholds of the fingers have a direct linear correlation with vibration exposure [7,8]. Beyond this, research has also shown that there is a latency where HAVS symptoms do not begin to manifest until around 2000 h of exposure, with some indication of symptoms evident in more than 50% of the exposed workers (in this case forestry workers) after 8000 h of exposure [9]. Therefore, by monitoring HTV exposure, the risk of serious injury can be reduced.

It has also been suggested that CTS, caused by the compression of the median nerve in the wrist, may be a result of significant HTV exposure in some cases. As highlighted in a review by Bovenzi [6] a selection of case studies showed a higher occurrence of CTS symptoms in occupations where vibrating tools were employed [10,11,12].

A seminal article by Griffin [13] highlighted the critical physical variables relevant to HTVs, these being: magnitude, frequency, duration, direction, area of contact, contact force, posture, and environment. Vibration analysis typically employs Fast-Fourier Transform (FFT) Vibration Spectrum Analysis (FFT VSA), as vibration induced injury is both frequency and amplitude dependent. HTV vibration levels are typically recorded as an acceleration value (in ms^−2^). A suitable transducer must therefore be sensitive over the correct frequency range for HTV monitoring, and sensitive to a range of amplitudes suitable to its application, which have been outlined in relevant standards [14,15]. Vibrations from power tools can occur in any axis of motion (depending on the tool) and therefore three-axis measurements are preferable.

The position of the vibration transducer is critical to the accuracy of HTV measurements as the maximum amplitude of the vibrations experienced by the power tool operator will likely be at the point of contact with the tool. This will normally be the palm of the hand, however the placement of a vibration transducer in this location may cause discomfort to the end user and impede their use of the tool. As such, when measurements are taken, these typically occur at the wrist [16], between the fingers [17] and on the machine itself [18]. Additionally, there is a commercially available transducer and glove solution that can be used to measure the vibration at the palm [19], however the transducer appears to be relatively large and heavy (the transducer and cable weigh 6 g [20]) and may affect the comfort of the user.

In this work, an innovative electronic textile solution for the monitoring of vibration has been created which allows for vibration transducers to be integrated within a glove at the palm of the hand and on the index finger without affecting the comfort of the end user. Electronic textiles have seen increasing development in recent years for sensing applications [21,22] leading to innovations including strain sensing [23], gas sensing [24], and pressure sensing [25].

A vibration-sensing electronic textile has been achieved by embedding small-scale tri-axial accelerometers into the core of a textile yarn using electronic yarn (E-yarn) technology. This resulted in a yarn with the look and feel of a normal textile. E-yarn technology has also been used to create other sensing devices such as temperature sensing E-yarns [26], acoustic sensing E-yarns [27], and light sensing E-yarns [28]. While this is not the first instance of accelerometers being used in electronic textiles [29,30] to the knowledge of the authors other works have not explored these E-textiles for vibration monitoring.

The production process to create the E-yarn, and the insertion of the E-yarn within a textile, has the potential to significantly dampen the vibrations and affect the sensors response. The E-yarns are constructed by first soldering the accelerometer onto fine copper wires. While the accelerometer is a commercially available device this is not a normal soldering practice (as instead the component would be attached to a printed circuit board). Further, not all of the solder pads are attached, and capacitors for bandwidth selection have not been used (the latter was to minimize the quantity of components needed within the E-yarn). This would likely result in the commercial device not providing an expected signal output. A similar approach was taken when developing the acoustic sensing E-yarn [27].

The soldered component is then encapsulated within a hard, resin, micro-pod to protect the mechanically fragile solder joints, help guard the component from external mechanical and chemical stress, and to attach supporting fibers. The inclusion of the resin micro-pod can have a significant effect on the response of an embedded sensor as has been observed for temperature sensing E-yarns [31] and optical sensing E-yarns [28], with the inclusion of the micro-pod influencing the nature of the measurands reaching the embedded component. While the micro-pod is hard, and good vibration transmission is likely, careful characterization of the embedded component is important to ensure that vibration transmission is achieved over all of the relevant frequencies, as resins are known to dampen the mechanical vibrations and that this dampening can be frequency dependent [32].

The encapsulated component, wires, and fibers are finally covered with an outer fibrous covering to consolidate the structure, facilitate further processing, and provide a normal textile feel. The inclusion of these soft fibers has previously been shown to affect the response of other sensors [28,31], and the use of textiles to dampen vibration is well reported in the literature [33,34,35]. The level of vibration dampening will likely be due to the structure and stiffness of the surrounding textile. This work will explore two covering designs, a knit-braid (as used in the majority of E-yarns to date) and a braid. Embedding the completed yarns within a textile fabric will have further potential to reduce the vibration transmission, and this must also be understood.

As each stage in the E-yarn production process could potentially introduce vibration dampening elements, this work carefully characterized the vibration-sensing E-yarns at each step during the production processes, and when embedded within a textile, over a range of vibration frequencies and amplitudes relevant to HTV monitoring.

This work builds on previous studies into the development of vibration-sensing E-yarns. The initial proof-of-concept vibration-sensing E-yarn utilized a slightly different design to either configuration shown in this work, and while it was capable of measuring vibrations, full characterization over relevant frequencies and amplitudes was not presented [36]. This article specifically expands a short proceeding previously presented by the authors [1] to include further data and a more comprehensive analysis, with this article introducing further characterization work of the E-yarn (amplitude dependence at different frequencies for the soldered and encapsulated components), additional repeatability data, the characterization of a different design of E-yarn, and the characterization of the E-yarns embedded within fabrics. Expanded experimental details have also been provided.

Ultimately this innovation has allowed a proof-of-concept vibration-sensing glove to be developed which incorporated four vibration-sensing E-yarns allowing for 3-axis vibration measurements to be taken at either the palm or the end of the middle figure; however the technology would also allow further vibration-sensing E-yarns to be integrated into a glove. This has the potential to providing a powerful new tool for understanding HTV and a possible solution for HTV monitoring in the workplace.

## 2. Materials and Methods

### 2.1. Vibration-Sensing E-Yarn Fabrication

**Figure 1:**
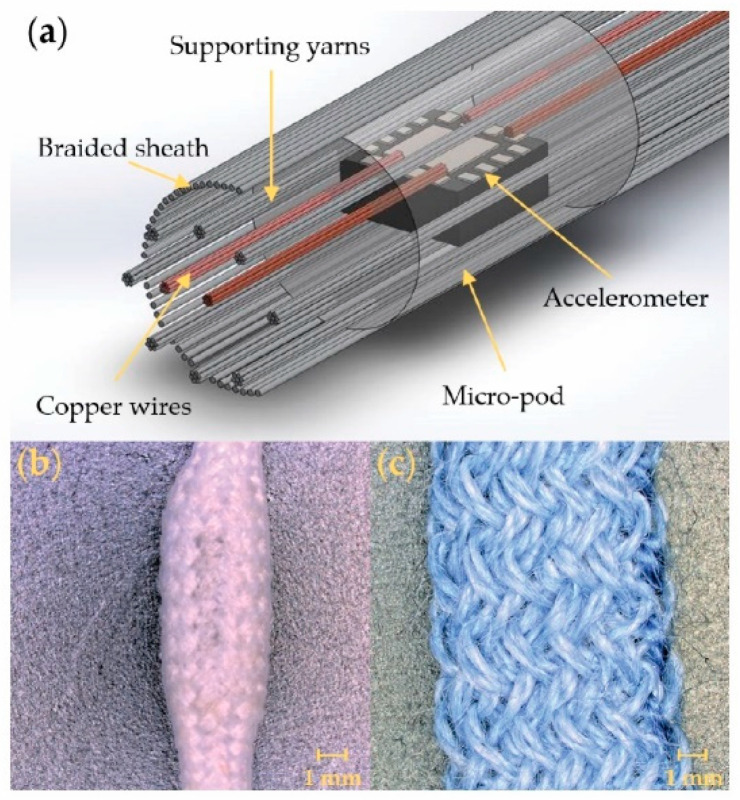
The vibration-sensing E-yarns. (**a**) Schematic of the final vibration-sensing E-yarn. (**b**) The final vibration-sensing E-yarn created using a braided outer sheath. (**c**) Vibration-sensing E-yarn constructed with a knit-braided outer sheath.

Most of the vibration-sensing E-yarns explored in this work used the design presented in the schematic shown in Figure 1a, and have previously been described elsewhere [1]. The vibration-sensing E-yarns were constructed in three discrete stages, as explained in the introduction. Initially an analogue MEMS (Micro-electromechanical systems) accelerometer (ADXL337; Analog Devices, Norwood, MA, USA) was soldered onto two multi-filament copper wires creating four solder joints corresponding to the x-axis and z-axis outputs, the power (voltage in), and the ground; the two soldered copper wires were cut in two locations with a scalpel to create the discrete electrical connections. The wires were arranged so that two copper wires left each end of the yarn (power and z-axis on one side, ground and z-axis on the other). The power and ground were connected using insulated copper wires which had seven enameled copper strands and was covered in a nylon sheath with a final diameter of 254 µm (BXL2001, OSCO Ltd. Milton Keynes, UK); the use of this wire electrically isolate the power and ground from the x-axis and z-axis outputs. The x-axis and z-axis outputs were connected using a seven-strand copper wire an overall diameter of 80 µm (strand diameter = 50 µm; KnightWire, Potters Bar, UK). While preliminary work had created an E-yarn where the y-axis of the commercial accelerometer was also connected [36], this was not connected in this work due to difficulties in reliably fabricating E-yarns with the y-axis output attached. This was because the y-axis solder pad was laid perpendicular to the other solder pads. Further, attaching this additional wire resulted in a wider micro-pod, and therefore wider E-yarn, which was undesirable.

The soldered accelerometer was then encapsulated within a resin micro-pod (crafted using Dymax 9001-E-V3.7; Dymax, Corporation, Torrington, CT, USA). The micro-pod also included eight textured, multifilament, polyester yarns (36 f/167 dtex; Ashworth and Sons, Cheshire, UK) that ran parallel to the copper wires. 

To complete the vibration-sensing E-yarn the encapsulated accelerometer, copper wires, and supporting fibers were inserted into a suture braiding machine (lay length = 5; RU1/24-80, Herzog GmbH, Oldenburg, Germany) and covered with a braided structure constructed from 24 carriers with polyester yarns (36 f/167 dtex; Ashworth and Sons, Cheshire, UK). This resulted in a final E-yarn with a 3.24 mm diameter that was mechanically robust and had the appearance of a normal textile yarn (see Figure 1b). A 0.325 m E-yarn weighed 0.6 g making it significantly lighter than the HAVS transducer.

To fully understand the design rules for the vibration-sensing E-yarns, experiments also explored using an alternative E-yarn design that used a looser and more open knit braided covering. This was similar to the covering used for a vibration-sensing E-yarn previously described in the literature [36] (however it should be highlighted that this preliminary design differed from what is presented here) and most other E-yarns presented to date [27,28,31]. To create the vibration-sensing E-yarns with a knit braid outer covering the copper-wire-micro-pod filament (constructed as described above) was inserted into a RIUS MC braiding machine (cylinder diameter = 8mm, 12 needles; RIUS, Barcelona, Spain) which created a circular warp-knitted structure (outer yarn: Folco 2/25NM F71 Composition: 50% (WV) Virgin Wool/50% (PC) Dralon^®^) over the filament using 12 additional polyester yarns (167 dtex/48 filaments). This resulted in a yarn with a final diameter of 7.74 mm, as shown in Figure 1c.

### 2.2. Inducing and Recording Vibrations

Vibrations were induced in this work using a Brüel & Kjær Type 4810 small vibration stage (Brüel & Kjær, Nærum, Denmark) which was driven by a power amplifier (Type 2718; Brüel & Kjær). The amplitude and frequency of the vibration level was monitored using a calibrated, tri-axial accelerometer (model number: 4520-001; Brüel & Kjær), and a signal analyzer (Photon+; Brüel & Kjær). Both the vibration stage and tri-axial accelerometer were controlled using the RT Pro Photon software (v 7.30.10; Brüel & Kjær, Nærum, Denmark). The signal analyzer and RT Pro Photon software were also used to collect and process signals from the vibration-sensing E-yarns.

**Figure 2:**
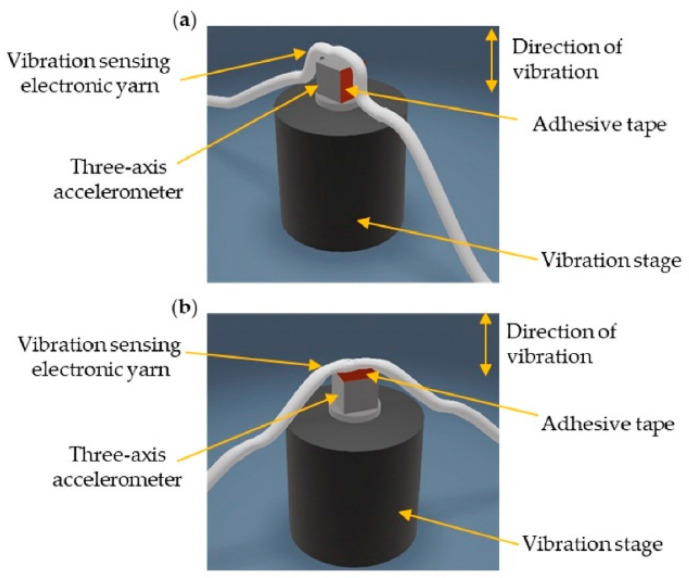
Schematic of the experimental set-up of the vibration-sensing E-yarn connected to the vibration stage. (**a**) Orientated for testing the x-axis response of the E-yarn. (**b**) Orientated for testing the z-axis response of the E-yarn.

**Figure A1:**
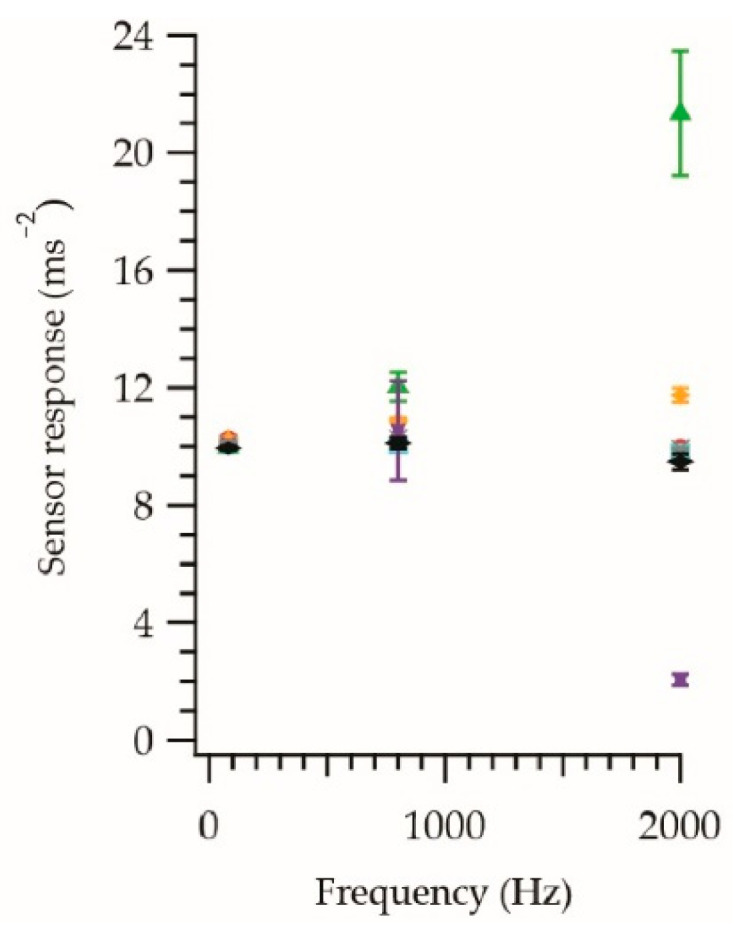
Accelerometer response when attached to the vibration stage using the screw 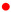, Tesa 51571 white double-sided cloth tape 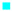, 3M 4026 white foam tape 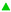, 3M 9088 transparent double sided plastic tape 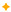, 3M scotch 9536 white foam tape 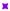, 3M 4910F VHB™ clear foam tape 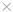 and beeswax 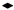 at different frequencies with a vibration amplitude of 10 ms^−2^.

For testing, the vibration-sensing E-yarns were securely attached to the tri-axial accelerometer, which was fixed to the top of the vibration stage (see Figure 2) using an adhesive tape. Force was applied to the top of the E-yarn to ensure consistent adhesion. It was critical to ensure that the inclusion of the tape did not affect the transmission of vibration between the E-yarn and the accelerometer, and therefore a short investigation using a selection of different tapes was conducted (see Appendix A): Tapes with a range of thicknesses (160–1600 µm) and adhesive properties (7.2–34 Ncm^−1^) were investigated. Ultimately, some tapes did influence the transmission of vibration over a frequency range of 8–2000 Hz, which was the range of interest in this study (see Figure A1). Therefore, tesa^®^ white, double-sided cloth tape (product number: 51571; tesa^®^ SE, Hamburg, Germany) was used throughout the work as it did not influence the transmitted vibration. The tape was replaced between each removal of the E-yarn to ensure a consistent level of adhesion. It was found that the tesa^®^ tape could easily be removed without leaving residue, so the vibration stage was not cleaned between experiments. 

As the vibration stage primarily vibrated in a single orientation, the E-yarn was re-orientated with respect to the motion of the vibration stage to calibrate the E-yarn in each of its axes (see Figure 2). It was observed that the embedded accelerometer gave different response dependent on which facing of the E-yarn was attached to the vibration stage (see Table A1). Consequently, the same side of the accelerometer needed to be identified once it was in the E-yarn form prior to conducting characterization experiments. The top facing (opposite facing to the solder pads) was attached to the vibration stage for all experiments.

**Figure A2:**
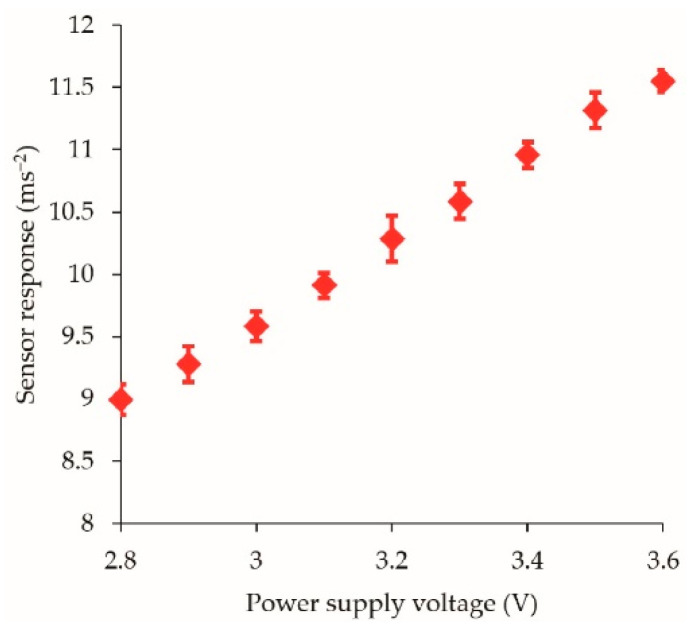
Graph of the relationship between the sensor response and the power supply voltage at a vibration amplitude of 10 ms^−2^ and at 80 Hz.

Power was supplied to the E-yarns using a benchtop variable power supply, with the E-yarns powered by 3.6 V in all cases (ISO-TECH IPS3303 Digital Bench Power Supply, ISO-TECH, Southport, UK). It should be noted that changing the supply voltage affected the sensors response (see Figure A2).

The response of the sensor to different frequencies and amplitudes of vibration were investigated in both the z-axis and x-axis orientations. For frequency dependence experiments a fixed amplitude of 1 ms^−2^ was used with six frequencies: 8 Hz, 80 Hz, 125 Hz, 250 Hz, 800 Hz, 1250 Hz, and 2000 Hz. For the amplitude dependence experiments five different amplitudes (10 ms^−2^, 5 ms^−2^, 2.5 ms^−2^, 1 ms^−2^, and 0.5 ms^−2^) were measured at three different frequencies (80 Hz, 800 Hz, and 1250 Hz). The choice of frequencies explored were inspired by relevant international standards for monitoring hand transmitted vibrations [14]. The authors concede that the amplitudes of vibration tested will not cover all of the high amplitude vibrations induced by some tools.

### 2.3. Data Collection and Analysis

For the collection and processing of data the RT Pro Photon software used a multiple frame Hanning window FFT and 25,600 lines. Root mean square (RMS) values were recorded for the induced vibration and the vibration-sensing E-yarns. For measurements of the E-yarn the calibration factor, converting the recorded voltage (mV) into an acceleration value (ms^−2^), provided by the manufacturers of the embedded accelerometer was used (300 mV/g [37]). For the E-yarn characterization experiments four separate E-yarns were used and the presented data are the averages of the responses from these four yarns with the corresponding error bars given by the standard deviation of the measurements. Other measurements were taken using a single E-yarn with measurements repeated five times, with the data presented being the average of these repeats (unless otherwise stated). The data figures presented in the work presenting data, and data fittings (with errors), were prepared using IGOR Pro (Version 7.0.2.2; Wavemetrics, Lake Oswego, OR, USA). Coefficients of determination were also provided by IGOR Pro. 

It should be noted that a 50 Hz signal was present throughout the measurements, with a second smaller noise peak at 150 Hz. It was believed that this noise was due to the use of mains power for the glove, recording apparatus, and the vibration stage.

### 2.4. Vibration-Sensing E-Yarns Embedded within Fabrics

**Figure 3:**
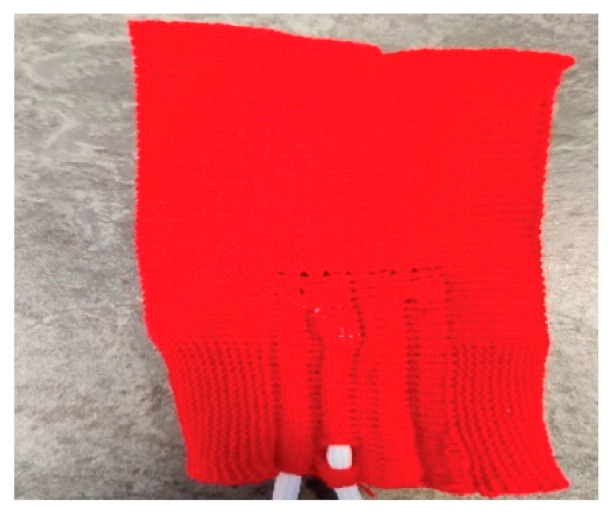
Image of a vibration-sensing E-yarn embedded into the testing fabric. This fabric was constructed using the same structure and materials of the final vibration-sensing glove.

Characterization of the E-yarns embedded within a glove was not practical using the available vibration stage given the weight and size of the glove, and the likelihood of poor adhesion between the glove and the vibration stage. Repeating the experiments using the same experimental set-up as for the E-yarns was important to facilitate a like-for-like comparison between the E-yarn before and after being embedded within the textile and to understand the effect of embedding accelerometers within yarns and then fabrics, which was the main purpose of this work. Therefore, once the glove was designed, a small sample fabric piece was produced using an identical knitted structure and materials used to produce the glove. This fabric is shown in Figure 3. As the fabric structure was identical to the structure used for the glove, characterizing the E-yarn within the fabric would allow for the response of the sensor when embedded within the glove to be understood. Both the fabric and glove were produced on a Shima Seiki SWG 091N 15 g knitting machine (Shima Seiki MFG., LTD; Wakayama-City, Japan), using Yeoman Yarns, Elastomeric 81% Nylon 19% Lycra (Yeoman Yarns, Leicester, UK). Both the fabric and the glove were seamless and comprised both purl knit and single jersey textiles. The fabrics had integrated tubes (channels) for insertion of the E-Yarns as shown in Figure 3.

## 3. Results

### 3.1. Characterzation of the Vibration-Sensing E-Yarns

#### 3.1.1. Vibration-Sensing E-Yarn Frequency Dependence

**Figure 4:**
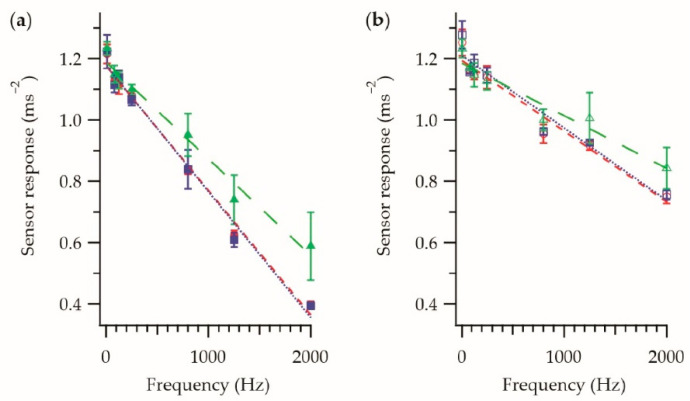
Frequency response of the accelerometer at different stages in the E-yarn manufacturing process. (**a**) Frequency response in the z-axis. Soldered stage 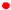; encapsulated stage 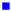; the final E-yarn 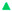. (**b**) The frequency response in the x-axis. Soldered stage 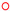; encapsulated stage 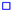; the final E-yarn 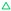. Each data point is an averaged measurement taken using four E-yarns, with the error bars representing the standard deviation in the measurements. Details of the linear fittings (dashed lines) are shown in Table 1. Previously presented elsewhere [1].

Initially, it was important to understand how the E-yarn production process affected the embedded sensors response. Figure 4 shows the frequency dependence of the accelerometer at each stage in the manufacturing process (soldered stage, encapsulated stage, final vibration-sensing E-yarn). 

Figure 4 shows that there was a linear relationship between the sensor response and the frequency in both the *z*-axis and *x*-axis, with the fitting parameters given in Table 1. It was unclear why there was a frequency dependence in the response, however this may have been attributed to the accelerometer being used in a different way than it was designed (i.e., to be attached to a printed circuit board), as the device was not soldered as intended (not all solder pads soldered, no filter capacitors), or could simply have been intrinsic to the accelerometer itself. The fact that the recorded sensor response exceeded 1 ms^−2^ at lower frequencies, while the platform was vibrating at that amplitude, further supported that the manufactures calibration value was not correct for the accelerometer when used in this way.

It was also observed from Figure 4, and the data fitting parameters shown in Table 1, that there was no significant difference in the sensor response between the soldered stage and the encapsulated stage; both the gradients and intercepts were in agreement within the error. This was due to the hard resin micro-pod (the micro-pod has a shore hardness of D45) providing a good medium for the transmission of vibration. A difference was observed once the braid was added. While at lower frequencies the experimental data for the final E-yarn was similar to the soldered and encapsulated stages (within the error), this was not the case at higher frequencies. The difference was likely due to the soft, deformable, fibers of the braid, and the structure of the braid itself, having a dampening effect on the vibrations. The relatively small errors, especially at lower frequencies, show that multiple (four) similar vibration-sensing E-yarns could be produced in a repeatable way.

#### 3.1.2. Vibration-Sensing E-Yarns Amplitude Dependence

**Figure 5:**
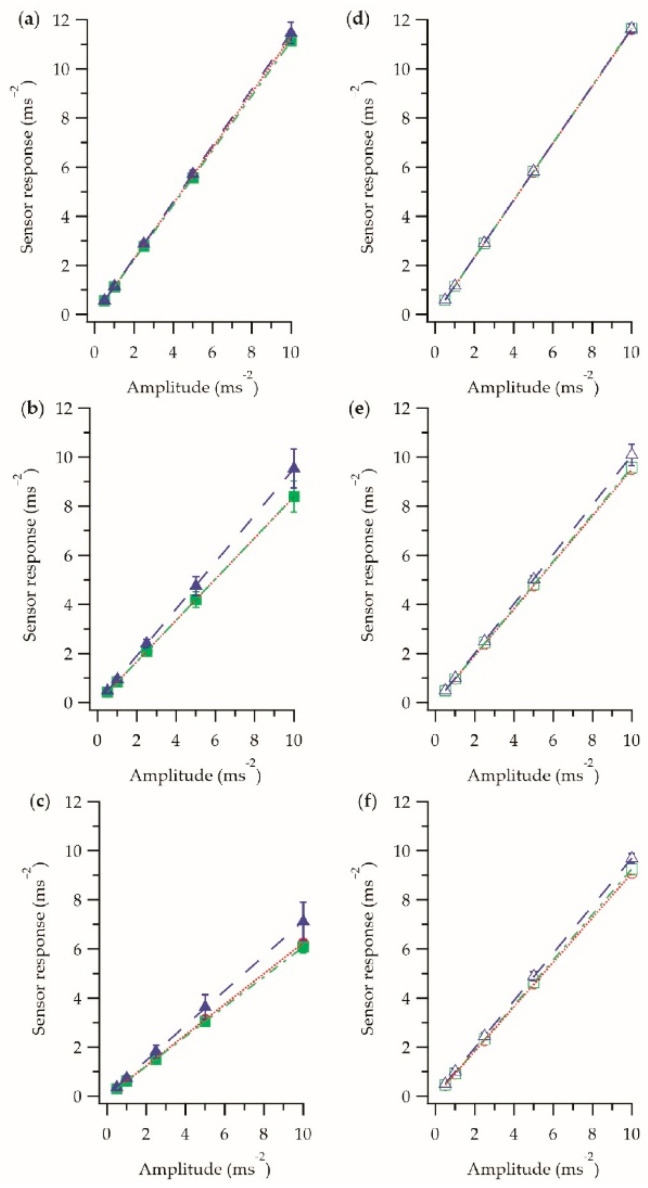
Sensor response of the E-yarns at different stages in the manufacturing process at three different frequencies. (**a**–**c**) Amplitude response in the *z*-axis at 80 Hz, 800 Hz and 1250 Hz respectively. Soldered stage 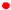; encapsulated stage 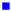; the final E-yarn 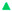. (**d**–**f**) Amplitude response in the x-axis at 80 Hz, 800 Hz and 1250 Hz respectively. Soldered stage 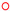; encapsulated stage 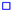; the final E-yarn 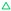. Each data point is an averaged measurement taken using four E-yarns, with the error bars representing the standard deviation in the measurements. Details of the linear fittings (dashed lines) are shown in Table 2. The results for the final E-yarn has previously been presented elsewhere [1].

The amplitude dependence for the vibration-sensing E-yarns at each stage in the production process at different frequencies was also investigated. Figure 5 shows the sensor response in the z-axis (Figure 5a–c) and x-axis (Figure 5d–f) at three different frequencies.

The results showed that there was a linear relationship between the sensor response and amplitude, which would be expected. This relationship was different for each frequency, which is in agreement with the data from Figure 4.

Figure 5a,d showed that at 80 Hz the manufacturing process has little effect on the sensor’s response as all of the experimental data were in close agreement. The actual gradients of the fittings were not in agreement (within error), suggesting that the error on the fitting may have been an underestimate.

At higher frequencies there was a more noticeable difference between the averaged response of the soldered accelerometer and encapsulated accelerometer, and the vibration-sensing E-yarn; however, the data were still largely in agreement within the errors. It was possible that the natural frequency of the knitted structure was between 800 and 1250 Hz, introducing some dampening at higher frequencies. Overall, Figure 5 showed that embedding an accelerometer within the structure of a yarn (to create an E-yarn) made very little difference to the accelerometer’s response. 

#### 3.1.3. Validating the Accuracy of the Vibration-Sensing E-Yarn Measurements

**Figure 6:**
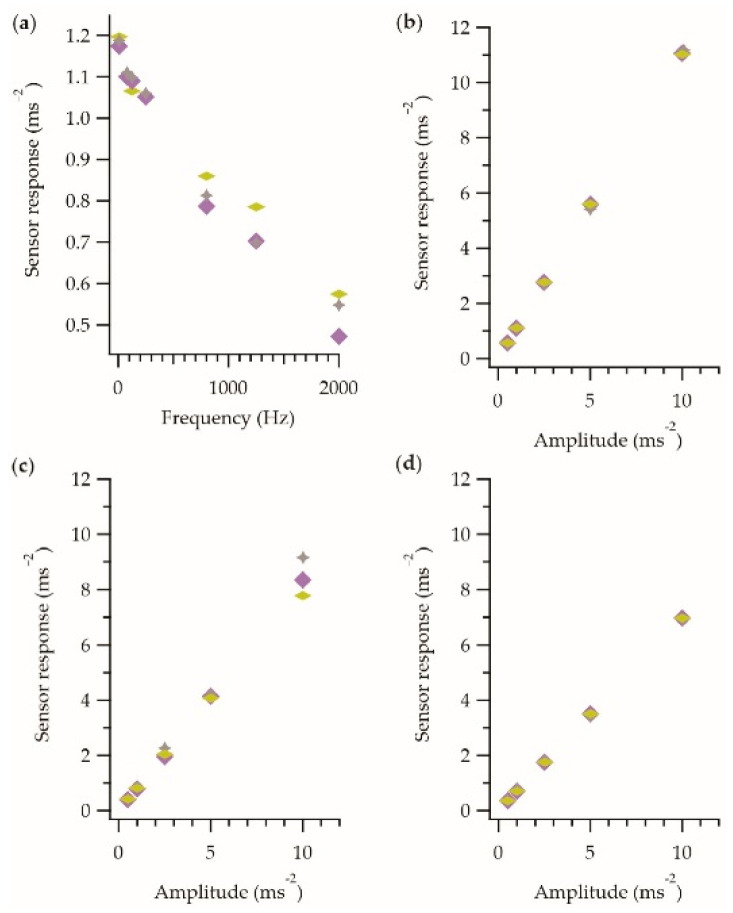
Repeat measurements taken using a single vibration-sensing E-yarn where the measurements were taken in different orders: sequential 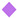 (as described in the methods section), reverse sequential 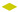 and random order 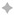. All data have been recorded in the z-axis only. (**a**) Frequency response data. (**b**) Amplitude response at 80 Hz. (**c**) Amplitude response at 800 Hz. (**d**) Amplitude response at 1250 Hz.

To confirm that the observed behavior was not an artefact of the measurement process, experiments were repeated with a single E-yarn with the frequency and amplitude measurements taken in a different order (as opposed to sequentially). This data also provided information about whether the observed variations between measurements shown in Figure 4 and Figure 5 were due to differences between the accelerometers (or E-yarns), or due to the intrinsic accuracy of the measurement process. Figure 6a shows frequency dependence data, while Figure 6b–d show the amplitude dependence for three different frequencies (80 Hz, 800 Hz, 1250 Hz).

The results in Figure 6 showed the expected linear trends for both the frequency and amplitude dependences, and importantly demonstrated that the measurement method did not significantly affect the results. Further, these results indicated that the variations in the measurements observed in Figure 4 and Figure 5 could be attributed to the measurement technique and not accelerometer/E-yarn variations. Measurement errors were likely introduced by factors such as variations in the placement of the yarn onto the vibration stage.

### 3.2. Constructing Vibration-Sensing E-Yarns Using a Knit Braided Outer Sheath

Most of the previous work using E-yarns have employed an outer sheath created using a knit braid structure. During preliminary experiments there was a concern that the poor fit of the micro-pod within the braid structure was leading to poor vibration transmission in some cases. Further, the relatively loose structure meant that during use the accelerometer could potentially move within the yarn, affecting its orientation. As such, this work focused on using a tight, braided structure to complete the vibration-sensing E-yarns.

**Figure 7:**
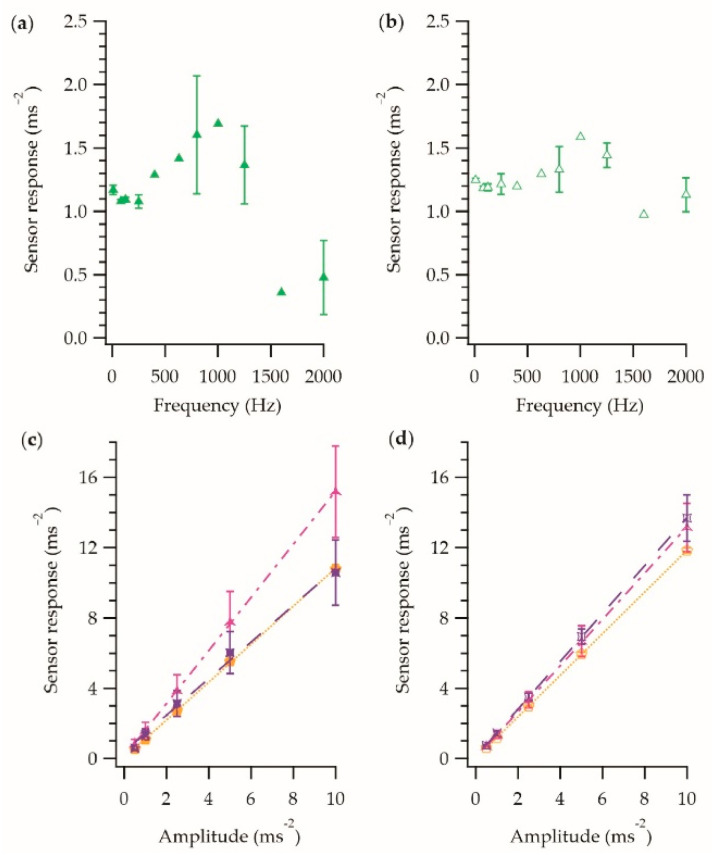
Sensor response of a vibration-sensing E-yarn constructed with a large diameter knit braid as the outer sheath. (**a**) Frequency response in the z-axis. (**b**) Frequency response in the *x*-axis. (**c**) The amplitude response for the different frequencies in the *z*-axis: 80 Hz 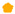; 800 Hz 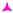; 1250 Hz 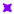. (**d**) The amplitude response for the different frequencies in the x-axis: 80 Hz 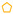; 800 Hz 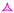; 1250 Hz 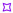. Each data point is the average of four measurements taken using a single E-yarn, with the error bars representing the standard deviation in the measurements.

For completeness, Figure 7 shows the behavior of a vibration-sensing E-yarn constructed using a large-diameter knit-braid. Figure 7a,b shows the frequency dependence of the sensor response in the *z*-axis and *x*-axis respectively. Figure 7c,d shows the amplitude dependence of the sensor response in the z-axis and x-axis respectively for three different frequencies.

From Figure 7, it was clear that the vibration-sensing E-yarn made with the large knit braid behaved differently to the other vibration-sensing E-yarns shown in this work. It was observed that the amplitude response (Figure 7c,d) showed a similar general linear relationship to what had been observed for the vibration-sensing E-yarn that was constructed using a braided outer sheath, however the fitting parameters differed, especially at higher frequencies (see Table 3), showing the greater dampening affect due to this knitted structure. The errors shown, especially in the z-axis, were larger than those previously observed, suggesting movement of the encapsulated component within the E-yarn’s structure. The frequency response (Figure 7a,b) did not have the same linear relationship previously observed when an outer sheath constructed using a knit braided was employed. This relationship (especially in the case of the *z*-axis) would make extracting meaningful data from the vibration-sensing yarn difficult in a real-life scenario.

The data in Figure 7 helped highlight an important design consideration for textiles constructed using the vibration-sensing E-yarns. When a lose structure was used the response of the E-yarn at different frequencies was not linear, and the errors in the measurements were greater when compared to using a tight structure. This suggested that the fabric structure that the E-yarns are incorporated into to create vibration-sensing fabrics must also be tight to minimize the measurement errors and facilitate easier interruption of the acquired data.

### 3.3. Characterisation of Vibration-Sensing E-Yarns Embedded within Fabrics

**Figure 8:**
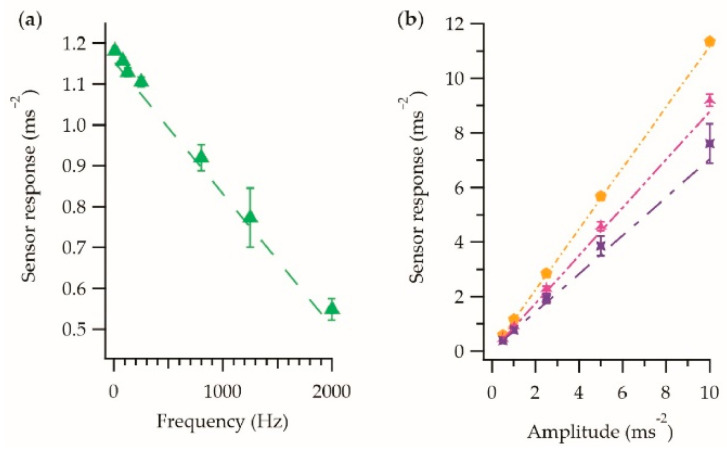
Sensor response of a vibration-sensing E-yarn embedded into the fabric. (**a**) Frequency response in the *z*-axis. (**b**) The amplitude response for the different frequencies in the *z*-axis. 80 Hz 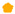; 800 Hz 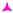; 1250 Hz 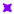. Each data point is the average of five measurements taken using a single E-yarn, with the error bars representing the standard deviation in the measurements.

It was understood that the vibration-sensing E-yarn had to be incorporated into a tight-fitting fabric (as discussed in Section 3.2), and this was used to inform the design of the vibration-sensing glove. Once the glove had been designed, a sample fabric using the same structure and materials of the glove was produced. Figure 8 shows the behavior of the vibration-sensing E-yarn when embedded within the fabric sample: The measurements were taken in the z-axis only as it is not physically possible to attach the fabric to the vibration stage in the x-axis.

Figure 8 showed the same linear relationships that were previously observed in Figure 4 and Figure 5. Table 4 shows the fitting parameters for the frequency and amplitude responses; these fitting parameters were similar to those presented in Table 1 and Table 2 for the vibration-sensing E-yarn. The frequency dependence was in agreement with the E-yarn data within the error. While not in agreement within the error, the data fittings for the amplitude dependency were still very close to the E-yarn data (for example, the fabric dataset fitting gradient at 80 Hz was 1.13 ± 0.47 × 10^−3^ compared to 1.15 ± 0.92 × 10^−3^ for the E-yarn). Practically, experimental data collected using the E-yarn embedded within a fabric would still be in agreement with the E-yarn fittings within their experimental error. The disagreement between the data fittings is likely an underestimation of the error on the fitting.

**Figure 9:**
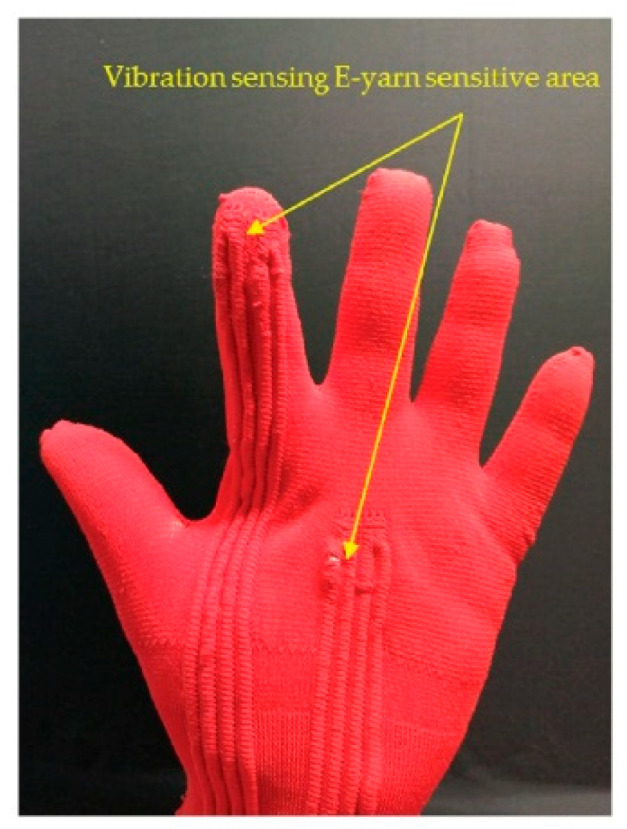
Photograph of the prototype vibration-sensing glove. The glove incorporates four vibration-sensing E-yarns, two at the palm, and two on the index finger.

This understanding allowed for the final vibration-sensing glove to be created. The glove incorporated four vibration-sensing E-yarns: two at the finger and two on the palm (Figure 9). The two yarns at each sensing site were orientated so that the x-axis of the embedded sensors were perpendicular to one another allowing for y-axis measurements to be taken (each site can take two z-axis measurements, an x-axis measurement, and a y-axis measurement).

## 4. Conclusions

This work presented a vibration-sensing E-yarn that would be suitable for monitoring HTVs. The vibration-sensing E-yarns were carefully characterized over a series of vibration amplitudes and frequencies that may be relevant to HTV monitoring. The development of this yarn allowed for a vibration-sensing glove to be engineered.

It was observed that the incorporation of small MEMS accelerometers within the structure of a textile yarn had little effect on its behavior, suggesting that the micro-pod encapsulation and braided structure did not significantly absorb vibrations. A linear relationship between the vibration-sensing E-yarn’s response and vibration amplitude at the different frequencies was confirmed. A linear relationship was also observed between the vibration-sensing E-yarn’s response and the frequency of the vibration. These relationships could be used to calibrate the response from the vibration-sensing E-yarn to determine vibration in a real-world scenario. Multiple yarns were compared showing that each behaved in a similar fashion.

This work also showed an alternative vibration-sensing E-yarn design that utilized a knit-braided outer sheath. This design introduced more experimental errors, and the more complex relationship between the sensor response and frequency that was observed would make extracting meaningful data in a real-world scenario difficult. It was believed that these differences were due to the looser structure of the knit-braid in this vibration-sensing E-yarn’s design, which allowed the vibration-sensing element to move within the structure of the outer yarn. This informed the textile structure and materials used for the final vibration-sensing glove. This also highlighted the importance of the E-yarn design for correct transmission of vibration to the embedded component.

Finally, a fabric sample with an embedded vibration-sensing E-yarn was characterized, showing that the fabric had no effect on the E-yarns response. This allowed for a vibration-sensing glove to be constructed, which incorporated four vibration-sensing E-yarns.

While this proof-of-concept is an important step toward developing a glove to monitor HTV, further work will be needed to mature this device into either a viable health monitoring or research tool. Initially, specific use cases should be identified, and the E-yarns should be validated for the specific frequencies and amplitudes output by these tools. Depending on the amplitudes experienced this may require the accelerometer used to be changed. A comprehensive user trail would then be necessary to validate the glove. It is proposed that during such a trial the contact force exerted between the users hand and tool would also be recorded as this is known to be an important factor in vibration transmittance [13].

## Figures and Tables

**Table 1:** Fitting parameters for the frequency response experiments at the different stages of the manufacturing process. S is the sensor response, f is the frequency.

	Data Fitting	Coefficient of Determination (R^2^)
Z-axis; soldered	S = (−4.06 ± 0.18) × 10^−4^ f + 1.18 ± 0.02	0.99
Z-axis; encapsulated	S = (−4.11 ± 0.22) × 10^−4^ f + 1.18 ± 0.02	0.99
Z-axis; final E-yarn	S = (−3.15 ± 0.21) × 10^−4^ f + 1.19 ± 0.02	0.98
X-axis; soldered	S = (−2.31 ± 0.21) × 10^−4^ f + 1.20 ± 0.02	0.96
X-axis; encapsulated	S = (−2.37 ± 0.24) × 10^−4^ f + 1.21 ± 0.02	0.95
X-axis; final E-yarn	S = (−1.72 ± 0.20) × 10^−4^ f + 1.19 ± 0.02	0.94

**Table 2:** Fitting parameters for the amplitude response of the accelerometer at the different stages of the E-yarn manufacturing process. S is the sensor response, a is the amplitude.

	Frequency	Data Fitting	Coefficient of Determination (R^2^)
Z-axis; soldered	80 Hz	S = 1.13 ± 0.30 × 10^−3^ a + (1.85 ± 1.52) × 10^−3^	1.00
Z-axis; encapsulated	80 Hz	S = 1.11 ± 0.91 × 10^−3^ a − (0.82 ± 4.70) × 10^−3^	1.00
Z-axis; final E-yarn	80 Hz	S = 1.15 ± 0.92 × 10^−3^ a + (3.98 ± 4.74) × 10^−3^	1.00
Z-axis; soldered	800 Hz	S = 0.84 ± 0.70 × 10^−3^ a − (1.16 ± 3.61) × 10^−3^	1.00
Z-axis; encapsulated	800 Hz	S = 0.84 ± 0.37 × 10^−3^ a − (1.08 ± 1.89) × 10^3^	1.00
Z-axis; final E-yarn	800 Hz	S = 0.95 ± 1.77 × 10^−3^ a + (5.13 ± 9.10) × 10^−3^	1.00
Z-axis; soldered	1250 Hz	S = 0.62 ± 0.27 × 10^−3^ a + (2.67 ± 1.38) × 10^3^	1.00
Z-axis; encapsulated	1250 Hz	S = 0.61 ± 0.32 × 10^−3^ a + (0.92 ± 1.65) × 10^−3^	1.00
Z-axis; final E-yarn	1250 Hz	S = 0.71 ± 4.60 × 10^−3^ a + (3.74 ± 23.70) × 10^−3^	1.00
X-axis; soldered	80 Hz	S = 1.16 ± 0.53 × 10^−3^ a + (4.12 ± 2.72) × 10^−3^	1.00
X-axis; encapsulated	80 Hz	S = 1.17 ± 0.88 × 10^−3^ a − (6.82 ± 4.53) × 10^−3^	1.00
X-axis; final E-yarn	80 Hz	S = 1.16 ± 0.35 × 10^−3^ a + (8.50 ± 1.79) × 10^−3^	1.00
X-axis; soldered	800 Hz	S = 0.95 ± 1.40 × 10^−3^ a + (16.95 ± 7.22) × 10^−3^	1.00
X-axis; encapsulated	800 Hz	S = 0.96 ± 3.18 × 10^−3^ a + (20.22 ± 16.40) × 10^−3^	1.00
X-axis; final E-yarn	800 Hz	S = 1.01 ± 1.3 × 10^−3^ a + (12.70 ± 6.71) × 10^−3^	1.00
X-axis; soldered	1250 Hz	S = 0.91 ± 1.90 × 10^−3^ a + (13.73 ± 9.78) × 10^−3^	1.00
X-axis; encapsulated	1250 Hz	S = 0.92 ± 1.03 × 10^−3^ a + (6.92 ± 5.28) × 10^−3^	1.00
X-axis; final E-yarn	1250 Hz	S = 0.97 ± 1.84 × 10^−3^ a + (37.60 ± 9.46) × 10^−3^	1.00

**Table 3:** Fitting parameters for the amplitude response of the vibration-sensing E-yarn created with a large knit-braided outer sheath. S is the sensor response, a is the amplitude.

Axis	Frequency	Data Fitting	Coefficient of Determination (R^2^)
Z	80 Hz	S = 1.09 ± 9.66 × 10^−3^ a + 0.03 ± 0.05	1.00
Z	800 Hz	S = 1.51 ± 9.19 × 10^−3^ a + 0.10 ± 0.05	1.00
Z	1250 Hz	S = 1.04 ± 45.00 × 10^−3^ a + 0.38 ± 0.23	1.00
X	80 Hz	S = 1.19 ± 2.24 × 10^−3^ a + 0.01 ± 0.01	1.00
X	800 Hz	S = 1.31 ± 6.19 × 10^−3^ a + 0.03 ± 0.32	1.00
X	1250 Hz	S = 1.36 ± 5.37 × 10^−3^ a + 0.09 ± 0.03	0.99

**Table 4:** Fitting parameters for the frequency and amplitude response of the vibration-sensing E-yarn embedded into fabric. S is the sensor response, f is the frequency, a is the amplitude.

	Data Fitting	Coefficient of Determination (R^2^)
Frequency dependence	S = (−3.18 ± 0.04) × 10^−4^ f + 1.18 ± 0.00	1.00
Amplitude dependence: 80 Hz	S = 1.13 ± 0.47 × 10^−3^ f + (21.02 ± 2.40) × 10^−3^	1.00
Amplitude dependence: 800 Hz	S = 0.92 ± 1.00 × 10^−3^ a − (3.85 ± 5.14) × 10^−3^	1.00
Amplitude dependence: 1250 Hz	S = 0.76 ± 2.79 × 10^−3^ a + (23.14 ± 14.40) × 10^−3^	1.00

## Data Availability

The data generated and analyzed during this study are included in this article. Raw data files used to generate the figures shown in this work are available on request from the corresponding author.

## Appendix A

### Appendix A.1. Experiments to Determine How to Attach the E-Yarn to the Vibration Stage

Experiments where performed to ensure that the method of attaching the E-yarn onto the accelerometer on the vibration stage would not interfere with the measured response from the E-yarn. To achieve this experiments were conducted by affixing the calibrated, commercially available accelerometer onto the vibration stage using different methods: The screw that came with the system to attach the accelerometer to the vibration stage, five double-sided tapes, and beeswax where tested. The five tested tapes were selected to provide a wide range of thicknesses and adhesive properties; Tesa 51,571 white double-sided cloth tape (thickness = 160 μm, adhesive properties = 12 Ncm^−1^ to steel; Tesa SE, Norderstedt, Germany), 3M 4026 White Foam Tape (thickness = 1600 µm, adhesive properties = 27.5 Ncm^−1^; 3M, Minnesota, MN, USA), 3M 9088 transparent double sided plastic tape (thickness = 210 µm, adhesive properties = 15 Ncm^−1^), 3M scotch 9536 white foam tape (thickness = 1100 µm, adhesive properties = 17 Ncm^−1^), 3M 4910F VHB™ clear foam tape (thickness = 1000 µm, adhesive properties = 26 Ncm^−1^).

Measurements were taken with a vibration amplitude of 10 ms^−2^ and three frequencies (80 Hz, 800 Hz, and 2000 Hz). The amplitude for vibration was set using readings where the accelerometer was screwed onto the vibration stage (therefore, these values were always 10 ms^−2^). Once this level of vibration was set, the screw was removed and the adhesive (tape or beeswax) was used to attach the accelerometer to the vibration stage. Figure A1 shows the response of the accelerometer using the different attachment methods at 10 ms^−2^ at three different frequencies.

Figure A1 shows that the Tesa 51,571 white double-sided cloth tape, 3M 4910F VHB™ clear foam tape, and the beeswax gave the most similar results at all of the frequencies tested. The beeswax required much more energy to make it stick to the vibration stage and the fabric E-yarn would not have stuck well to it. Attaching the accelerometer with the Tesa 51,571 white double-sided cloth tape gave the best results. Both the 3M 4026 white foam tape and the 3M scotch 9536 white foam tape interfered with the response of the accelerometer at the higher frequency tested. The use of the 3M 9088 transparent double sided plastic tape also slightly affected the sensor’s response at the highest frequency tested.

### Appendix A.2. E-Yarn Facing Experiments

At the soldered and encapsulated stages, the E-yarn was measured with the soldered pads facing up. This was kept consistent for all of the experiments. At the final E-yarn stage, the preliminary results were much more variable. Therefore, an encapsulated E-yarn was measured in different directions, with the solder pads facing down when attached to the vibration stage, and with the solder pads facing left and right (i.e., with the accelerometer on either side). Table A1 shows that the sensor response in each direction when subjected to a 10 ms^−2^, 80 Hz vibration in the z-axis. These measurements were taken five times and the average was recorded as shown in Table A1.

**Table A1:** Sensor response when attached to the vibration stage at different positions.

Direction	Vibration Amplitude (ms^−2^)	Sensor Response (ms^−2^)
Up	10.09 ± 0.03	10.66 ± 0.03
Down	10.14 ± 0.02	7.22 ± 0.01
Left	9.82 ± 0.02	1.04 ± 0.15
Right	10.18 ± 0.01	4.93 ± 0.51

As a result of the findings in Table A1 the facing of the E-yarn was carefully checked before characterization experiments were conducted.

### Appendix A.3. Applied Voltage Experiments

The datasheet for the ADXL337 showed that the required input power for the accelerometer was 3 V to 3.6 V. However, it was observed that lowing the voltage applied reduced the sensor response (when the accelerometer was soldered as described earlier). Figure A2 shows the relationship between the power supply voltage and the sensor response. The induced vibration was at 10 ms^−2^ and 80 Hz for these experiments.

Figure A2 shows there is a linear relationship between the power supply voltage and the sensor response. This is most likely due to the way that the sensor was soldered to create the E-yarn. This graph has been included to facilitate replication of these experiments.
